# The effects of verbal information and approach-avoidance training on children's fear-related responses

**DOI:** 10.1016/j.jbtep.2015.01.008

**Published:** 2015-09

**Authors:** Kathryn J. Lester, Stephen C. Lisk, Nina Mikita, Sophie Mitchell, Jorg Huijding, Mike Rinck, Andy P. Field

**Affiliations:** a MRC Social, Genetic and Developmental Psychiatry Centre, Institute of Psychiatry, Psychology and Neuroscience, King's College London, De Crespigny Park, London SE5 8AF, UK; b School of Psychology, University of Sussex, Pevensey Building, Falmer, Brighton BN1 9QH, UK; c Institute of Psychology, Erasmus University Rotterdam, Woudestein T13, Postbus 1738, 3000 DR Rotterdam, The Netherlands; d Behavioural Science Institute, Radboud University Nijmegen, PO Box 9104, 6500 HE Nijmegen, The Netherlands

**Keywords:** Fear, Children, Approach-avoidance, Verbal information

## Abstract

**Background and objectives:**

This study examined the effects of verbal information and approach-avoidance training on fear-related cognitive and behavioural responses about novel animals.

**Methods:**

One hundred and sixty children (7–11 years) were randomly allocated to receive: a) positive verbal information about one novel animal and threat information about a second novel animal (verbal information condition); b) approach-avoidance training in which they repeatedly pushed away (avoid) or pulled closer (approach) pictures of the animals (approach-avoidance training), c) a combined condition in which verbal information was given prior to approach-avoidance training (verbal information + approach-avoidance training) and d) a combined condition in which approach-avoidance training was given prior to verbal information (approach-avoidance training + verbal information).

**Results:**

Threat and positive information significantly increased and decreased fear beliefs and avoidance behaviour respectively. Approach-avoidance training was successful in training the desired behavioural responses but had limited effects on fear-related responses. Verbal information and both combined conditions resulted in significantly larger effects than approach-avoidance training. We found no evidence for an additive effect of these pathways.

**Limitations:**

This study used a non-clinical sample and focused on novel animals rather than animals about which children already had experience or established fears. The study also compared positive information/approach with threat information/avoid training, limiting specific conclusions regarding the independent effects of these conditions.

**Conclusions:**

The present study finds little evidence in support of a possible causal role for behavioural response training in the aetiology of childhood fear. However, the provision of verbal information appears to be an important pathway involved in the aetiology of childhood fear.

## Highlights

• Verbal information and approach-avoidance behavioural training are compared.
• Threat information increases and positive information decreases fear responses.
• Training approach and avoidance behaviours has limited effects on fear responses.
• No significant additive effects of these pathways to fear in children were observed.
• Verbal information is an important pathway in the aetiology of childhood fear.

## 1 Introduction

Childhood fear and anxiety is highly prevalent (Cartwright-Hatton, McNicol, & Doubleday, 2006) with the mean age of onset for childhood anxiety disorders being 11 years of age (Kessler et al., 2005). Childhood anxiety often persists into adolescence and adulthood (Weems, 2008) and is associated with academic difficulties (Owens, Stevenson, Norgate, & Hadwin, 2008), impaired social functioning and peer difficulties (Asendorpf, Denissen, & van Aken, 2008; Erath, Flanagan, & Bierman, 2007), and is a major risk factor for subsequent psychological (Kim-Cohen et al., 2003) and physical health problems (Beesdo et al., 2009). Specific fears are common in childhood with children experiencing general patterns of normative fear throughout their development with animal fears typically emerging in middle childhood (Field & Davey, 2001). The majority of childhood fears will remit spontaneously, however, for a significant minority of children, their fear and anxiety persists. Research aimed at better elucidating the mechanisms involved in the aetiology of childhood fears and anxiety is especially important given the considerable suffering, dysfunction and poor prognosis associated with anxiety. This research also has the potential to inform the development and testing of novel prevention and treatment methods.

According to cognitive models of anxiety, exposure to a potentially threat-provoking stimulus activates both semantic-evaluative information and behavioural responses such as avoidance (Beck & Clark, 1997; Ouimet, Gawronski, & Dozois, 2009). Recent research has experimentally manipulated semantic evaluative information or behavioural responses to investigate the possible causal effects on fear-related cognitions and behaviours. One approach has been to investigate how fear develops in children given that many adult fears are rooted in childhood (Huijding et al., 2009; Muris, 2007).

Rachman's three pathways to fear model suggests that fears are acquired environmentally via a direct route of aversive classical conditioning and two indirect routes of modelling/vicarious learning through observing others and threatening semantic evaluative information transmission (Rachman, 1977). Empirical research supports both direct and indirect pathways to fear acquisition, although much of this evidence comes from retrospective studies thereby limiting conclusions regarding the causal role of these pathways in fear acquisition (Askew & Field, 2008; King, Eleonora, & Ollendick, 1998; Muris & Field, 2010). Fortunately, more recent research has used prospective paradigms in which participants are presented with threatening semantic evaluative information about a stimulus and the effect of this information on fear-related cognitions and behaviour established. Research with children has shown that providing verbal threat information about a novel animal (an Australian marsupial) increases children's fear beliefs (e.g. Field, Lawson, & Banerjee, 2008; Muris, van Zwol, Huijding, & Mayer, 2010), influences avoidance behaviour (e.g. Field & Lawson, 2003), biases selective attention and future contingency learning (Field, 2006b; Field & Lawson, 2008) and affects physiological responses (Field & Price-Evans, 2009; Field & Schorah, 2007). Providing positive information reduces children's fear beliefs and behavioural avoidance (Field & Lawson, 2003; Kelly, Barker, Field, Wilson, & Reynolds, 2010; Muris, Bodden, Merckelbach, Ollendick, & King, 2003).

In recent years, Approach-Avoidance Tasks (AAT) have been used to study behavioural responses in a range of fears (Heuer, Rinck, & Becker, 2007; Lange, Keijsers, Becker, & Rinck, 2008; Rinck & Becker, 2007; Roelofs et al., 2010). In the AAT, participants respond to a stimulus presented on screen by pulling it toward them or pushing it away using a joystick. The task builds on the premise that stimuli appraised as negative produce automatic avoidance tendencies characterised by pushing objects away from oneself, while stimuli appraised as pleasant produce automatic approach tendencies characterised by pulling the objects closer (Chen & Bargh, 1999; Solarz, 1960). These approach-avoidance tendencies have been found to differentiate high and low fearful individuals (Klein, Becker, & Rinck, 2011; Rinck & Becker, 2007; Roefs et al., 2011). For example, spider-fearful participants were faster to push spider pictures away, indicative of avoidance, than to pull them closer with this effect not observed in non-fearful participants (Rinck & Becker, 2007).

Using a modified approach-avoidance task it is also possible to experimentally manipulate behavioural response tendencies in order to begin to study the possible causal effect on fear-related cognitions and behaviours (for reviews see Vandenbosch & De Houwer, 2011; Woud, Becker, & Rinck, 2011). In a modified AAT, participants are trained to repeatedly pull toward (training approach tendencies) or push away (training avoidance) neutral stimuli using a joystick with the direction of response required determined by some characteristic of the stimuli (e.g. push brown pictures, pull blue pictures). The premise being that repeatedly training approach tendencies by pulling the stimulus closer is hypothesised to lead to more positive appraisals, while training avoidance tendencies would lead to more negative appraisals (Chen & Bargh, 1999; Solarz, 1960).

Two studies have investigated the effects of manipulating behavioural-response tendencies on fear-related responses about novel animals in children (Huijding et al., 2009; Huijding, Muris, Lester, Field, & Joosse, 2011). Children were instructed to repeatedly pull toward (approach) or push away (avoid) pictures of Australian marsupials, quokka and quoll. In the first study, the approached animal was rated more positively while disliking of the avoided animal increased (Huijding et al., 2009). Greater avoidance and fear of the avoided animal relative to the approached animal was reported in girls only. No significant effects were observed on a measure of implicit attitudes, the Implicit Association Test (IAT, Greenwald, McGhee, & Schwartz, 1998). In a second study (Huijding et al., 2011), training increased self-reported liking of the approached animal and disliking and fear of the avoided animal. Children sought out more negative information about the avoided animal and more positive information about the approached animal. Once again, no significant effects were observed on implicit attitudes.

Associative learning is one possible mechanism explaining how verbal information operates (Field, 2006a). Telling a child a dog is dangerous may evoke a mental representation of threat that becomes linked in memory with the concept of dogs (Muris & Field, 2010). Few studies have investigated interactions between possible pathways to fear learning. However, giving verbal threat information before a direct negative conditioning experience or vicarious learning episode yielded a magnified (additive) effect on fear beliefs compared to either pathway alone (Askew, Kessock-Philip, & Field, 2008; Field & Storksen-Coulson, 2007). These findings were interpreted as consistent with conditioning (association formation) models of anxiety (Davey, 1997). Expectancies about the possible outcomes (unconditioned stimulus (US)) of interacting with the animal (conditioned stimulus, CS) shaped future fear learning by strengthening the CS-US association formed during a negative conditioning/vicarious learning experience. Verbal information may be a powerful means through which the strength of a conditioned response (CR) elicited by a CS can be revalued or devalued through a process called US revaluation (Davey, 1997). However, presenting verbal information after a vicarious learning episode in an attempt to revalue the US did not significantly magnify changes in fear beliefs compared to vicarious learning alone (Askew et al., 2008, Exp 3).

Approach-avoidance training operates via association formation principles, namely operant (i.e. stimulus-response) evaluative conditioning (De Houwer, 2007; Woud, Becker, & Rinck, 2008). The animal picture acts as the discriminative stimulus (CS), which determines response (UR) (push or pull), in turn triggering the approach-avoidance system with a pull movement leading to a positive approach state (CR) and a push movement to a negative avoidance state, thus influencing fear-related responses (Woud et al., 2008). Association formation models predict that giving verbal information prior to approach-avoidance training would have an additive effect: Prior expectancies created via verbal information may act to strengthen the CS-UR association formed during approach-avoidance training leading to magnified fear effects. Likewise, presenting verbal information after approach-avoidance training may strengthen the CR thus magnifying the effects on fear-related responses. If a child receives avoidance training and then subsequently hears threat information about the same animal then they may revalue that animal as even more aversive.

Associative learning effects may also be driven through generating and evaluating propositions about relations in the environment, which in turn drives conditioned responding (De Houwer, 2009; Mitchell, De Houwer, & Lovibond, 2009). In line with a propositional account, verbal information may be sufficient to influence learning effects. Telling participants that the quoll is a dangerous animal is expected to create a proposition, which in turn drives fear-related responding. If it is proposition formation that drives conditioned responding and not association formation then we hypothesise that the addition of approach-avoidance training after verbal information is unlikely to have an additive effect on fear-related responses. Predictions regarding the impact of giving approach-avoidance training prior to verbal information are less clear from this model. In both instances the automatic link formation mechanism thought to underpin approach-avoidance training is non-propositional and therefore unlikely to be affected directly by verbal information.

In the present study, participants were assigned to verbal information (VI), approach avoidance training (AATT), verbal information then approach-avoidance training (VI + AATT) or approach-avoidance training then verbal information (AATT + VI). In an improvement to past experiments investigating the effect of approach-avoidance training, fear beliefs and indirect avoidance behaviour were assessed at pre- and post-time points permitting assessment of *change* in these measures. A direct measure of avoidance behaviour was included to test for effects on actual avoidance tendencies toward the novel animals. We hypothesise that repeated avoidance or verbal threat information would lead to an increase in fear beliefs, more negative implicit attitudes and greater avoidance behaviour. In contrast, repeated approach or verbal positive information was hypothesised to lead to a decrease in fear beliefs, more positive implicit attitudes and relatively less avoidance behaviour. We test competing hypotheses regarding propositional and association formation accounts by investigating the magnitude of fear-related effects across the four conditions (VI, AATT, VI + AATT and AATT + VI).

## 2 Method

### 2.1 Participants

One hundred and sixty children (78 males, 82 females, *M*_age_ = 8.94, *SD* = 1.13 years, range 7–11 years) were recruited from primary schools. Written informed consent was obtained from parents and verbal assent from the child and ethical approval obtained from the University of Sussex School of Life Sciences Research Governance Committee (AFKL1208). Participants were randomly assigned to verbal information only (VI, *N* = 40), approach-avoidance training only (AATT, *N* = 40), verbal information then AATT (VI + AATT, *N* = 40) or AATT then verbal information (AATT + VI, *N* = 40). These groups were comparable on gender distributions and trait anxiety (see Table 1) but not age. The VI condition were significantly younger than the AATT + VI condition (8.57 vs. 9.34 yrs, *p* = .01). No statistical method is available which would satisfactorily redress and control for the difference in age between groups (see Miller & Chapman, 2001, for discussion of why an ANCOVA is not an appropriate solution). However, all outcome variables were uncorrelated with age.

### 2.2 Materials

#### 2.2.1 Trait anxiety inventory for children (Spielberger, Gorsuch, Lushene, Vagg, & Jacobs, 1973)

This scale comprises 20 items with a three-point Likert-type response scale (1 = *hardly ever* to 3 = *often*) and measures stable individual differences in anxiety proneness. Cronbach's alpha was α = .82 and the mean trait anxiety score was 35.89 (*SD* = 6.81, range 21–53). Only 3.1% of the sample had trait anxiety scores exceeding 1.96SD above the normative mean of 37.35 (Spielberger et al., 1973).

#### 2.2.2 Animals

Clearly labelled pictures of Australian marsupials, the quoll and the quokka were used. No participants reported any prior experience of the animals and thus had no prior fear expectations.

#### 2.2.3 Fear Beliefs Questionnaire (FBQ; Field & Lawson, 2003)

Eight questions, repeated twice, once for each animal were presented in a random order (e.g. would you be happy if you found a quokka/quoll in your garden?). Responses were scored 0 (*No, not at all*) to 4 (*Yes, definitely*) with several items reverse scored. A high score was consistent with having a higher fear belief. Internal consistency was good (pre α = .70 for quokkas and α = .73 for quolls and post both α = .97).

#### 2.2.4 Affective priming task (APT)

This task has previously proven to be sensitive to the effect of a vicarious learning paradigm using the same novel animals (Askew & Field, 2007). Each trial began with a central fixation cross, presented for 500 ms followed by a picture prime depicting either the quokka or quoll for 200 ms. After a 100 ms interval, a target word was displayed which was either nice (e.g. joy, pretty) or nasty (e.g. cry, sick), which the participants categorised by pressing a key labelled nice or nasty. Pressing the correct key activated the next trial, which began after a 2000 ms interval. There were four different prime-target combinations: Threat information/avoid animal prime – negative target word (threat congruent trial), threat information/avoid animal prime – positive target word (threat incongruent trial), positive information/approach animal prime – positive target word (non-threat congruent trial) and positive information/approach animal prime – negative target word (non-threat incongruent trial). The classification of the target word was expected to be faster on prime-target congruent trials (when the picture prime has the same valence for the participant as the target word) than on prime-target incongruent trials. Participants completed 12 practice trials and 72 test trials (18 per condition) presented randomly.

#### 2.2.5 Nature reserve task (NRT; Field & Storksen-Coulson, 2007)

This task comprised a green felt board (45 × 65 cm) with trees and bushes made of pipe cleaners, depicting a nature reserve in Australia. Participants imagined a visit to the nature reserve and were given a Lego figure that represented them (a girl figure for females, and a boy figure for males). A picture of the quoll was then placed in one corner of the board and the participants were asked to place the figure on the board where they would like to be when the quoll was there. The procedure was then repeated for the quokka.^1^ The distance (in mm) between the figure and the middle of the picture was taken as an indirect measure of avoidance.

#### 2.2.6 Behavioural avoidance test (BAT; Field & Lawson, 2003)

Participants were presented with two modified cardboard pet carriers, each with a hole at one end, covered with hessian fabric with a slit in the middle so that the child was able to place their hand inside without being able to see the contents. Each box was clearly labelled with one of the animal names. Participants were told that the boxes contained the animals, which were nocturnal so they should be sleeping. Unbeknown to the participants, each box contained a furry toy and straw.

Participants sat 2 m away from the pet carriers. They were invited to approach the first box (quoll) to stroke the animal. The reaction time (sec) to complete the task was used as the dependent variable and was measured from the end of the verbal instruction until the participant placed their hand in the box up to the wrist. In line with Field et al. (2008) if the participant had not approached the box within 15 s, it was assumed that they were unwilling to stroke the given animal and for ethical reasons the experimenter moved on. After completing the first box, participants were asked to return to the chair, and the task was repeated for the quokka box.

#### 2.2.7 Experimental fear manipulations

Participants received positive information or approach training about one animal and threat information or avoidance training about the second animal. The assignment of training valence (positive-threat, approach-avoid) to animal (quoll-quokka) was randomised with 80 children given positive information/approach training about the quokka and 80 children the quoll. In the combined groups, participants approached the same animal that they heard positive information about and avoided the same animal that they heard threat information about.

##### 2.2.7.1 Verbal information (Field & Lawson, 2003)

Two versions of a story matched for length and content were used differing only in valence (threat or positive) with identical versions created about the quokka and quoll. Participants received threat information about one animal and positive information about the second animal. Information type and the order of presentation of threat and positive information were determined at random. The information was presented through headphones.

##### 2.2.7.2 Approach-avoidance training task (AATT; Huijding et al., 2009)

The AATT was selected to be identical to that used by Huijding et al., (2009), which has previously proven capable of inducing changes in explicit measures of fear. Children were trained to avoid (push) one animal and approach (pull) the other animal. The task comprised two training phases and three manipulation checks. Participants responded to on screen pictures of quokkas and quolls by pulling or pushing the Logitech Attack 3 joystick. Pulling the joystick made the picture increase in size creating the impression of approach while pushing the joystick away made the picture decrease in size giving the impression of avoidance. During the test phases, pushing or pulling the joystick all the way forward or backward made the picture disappear. During the training phases, the picture only disappeared if the correct response was made, thus forcing participants to make the required response before proceeding. To begin the next trial participants returned the joystick to the middle position and pressed the fire button.

During the practice, a picture of a quokka was presented, the experimenter told the child it was a quokka and then asked the child to move the joystick forward or backward until the picture disappeared. This was repeated for the quoll. The main experimental procedure then began.

*Phase 1 (baseline assessment):* Children were presented with pictures of the animals and were asked to say aloud as fast as possible whether the picture depicted a quoll or a quokka. After identifying each picture, they pushed or pulled the joystick to make the picture disappear and to proceed to the next trial. The experimenter did not suggest any response direction. This phase consisted of 24 trials (12 pictures per animal) presented in a fixed random order. The purpose of this phase was to ensure the participants were able to discriminate between quokkas and quolls and to establish a baseline measure of the proportion of spontaneous push and pull preferences for each animal.

*Phase 2 (first training phase):* Children were instructed to push away quokkas and pull toward them quolls, or vice versa for children in the opposite condition. This phase consisted of 48 trials (24 per animal) presented in a fixed random order. Six pictures of each animal were used and presented on four occasions.

*Phase 3 (post-training assessment 1):* Children were again presented with pictures of the animals and were instructed to name the animal as quickly as possible before pushing or pulling the joystick to proceed to the next trial. The experimenter did not suggest any particular response direction. This phase served as a manipulation check to assess whether participants “spontaneously” proceeded to the next trial with the response directions that were trained in the previous phase. This phase comprised 12 trials (6 for each animal) and the pictures used were different from those used during the training phase.

*Phase 4 (second training phase):* This phase was identical to phase 2.

*Phase 5 (post-training assessment 2):* This phase was identical to phase 3.

### 2.3 Procedure

**Fig. 1:**
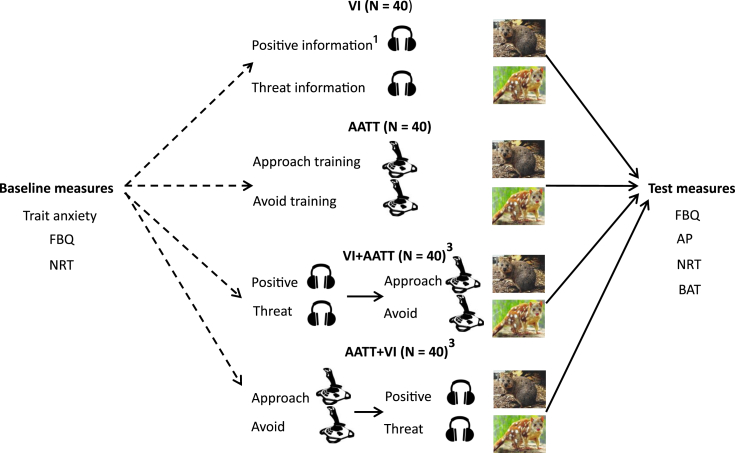
Experimental procedure. , random allocation of participants to groups; FBQ = Fear Beliefs Questionnaire; NRT = nature reserve task; APT = affective priming task; BAT = behavioural avoidance test. ^1^Order of administration of positive and threat information was randomised; ^2^Assignment of training valence (positive versus threat information and approach versus avoid training) to animal (quoll or quokka) was randomised; ^3^Participants in the combined conditions received positive information about the same animal that they received approach training and threat information about the animal that they received avoid training.

A research assistant saw each participant individually in a quiet classroom for approximately 50 min. Participants were told that the study was about their thoughts, feeling and behaviours around new animals. Participants began by completing a paper-pencil version of the trait anxiety scale. Participants were then introduced to quokka and quoll and the experimenter verified that the children had no prior knowledge of either animal. Participants then completed the FBQ followed by the NRT. Participants were then randomly allocated to complete one of the four experimental fear manipulations. Participants then repeated the FBQ, followed by the AP, the NRT and then the BAT (see Fig. 1). Participants were debriefed, told correct factual information about the animals and shown the true contents of the BAT boxes.

## 3 Results

### 3.1 Effects of approach-avoidance training on approach movements

The percentage of approach movements during the assessment phases was analysed using a repeated measures ANOVA with Animal (Approach, Avoid) and Phase (Baseline, Post 1, Post 2) as the within-participant and Group (AATT, VI + AATT, AATT + VI) as the between participant variable. More approach movements were made to the approach than avoid animal, *F*(1,117) = 123.53, *p* < .001, partial *η*^*2*^ = .51, 63% vs. 33%. Confirming that the training had the desired effect on responses, the Animal × Phase interaction attained significance, *F*(2,234) = 59.48, *p* < .001, partial *η*^*2*^ = 0.34. The 3-way interaction was non-significant, *F*(4,234) = 0.29*, p* = .87, *η*^*2*^ = 0.01.

**Fig. 2:**
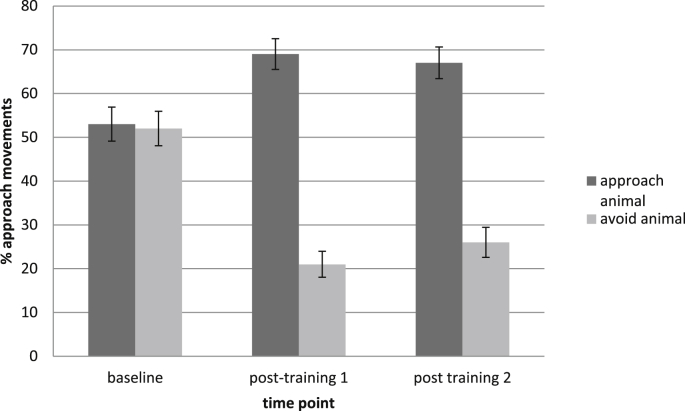
Percentage of approach movements at each time point (error bars ± 1 standard error).

For the approach animal, the percentage of approach movements increased significantly across time, Huynh-Feldt corrected *F*(1.82,216.35) = 12.14, *p* < .001, partial *η*^*2*^ = 0.09. The increase was significant between the baseline and first post-training phase, *F*(1,117) = 16.74, *p* < .001, partial *η*^*2*^ = 0.13, 53% vs. 69%, but not between the two post-training phases, *F*(1,117) = 0.90, *p* = .35, partial *η*^*2*^ = 0.01, 69% vs. 67%. For the avoid animal, the percentage of approach movements decreased significantly across time, Huynh-Feldt corrected *F*(1.82,216.70) = 41.28, *p* < .001, partial *η*^*2*^ = 0.26. The decrease was significant between the baseline and first post-training phase, *F*(1,117) = 63.31, *p* < .001, partial *η*^*2*^ = 0.35, 52% vs. 21%, but increased (non-significantly) between the two post-training phases, *F*(1,117) = 3.24, *p* = .08, partial *η*^*2*^ = 0.03, 21% vs. 26% (Fig. 2).

### 3.2 Effects on fear beliefs

A mixed ANOVA was performed on mean FBQ scores with Group (VI, AATT, VI + AATT, AATT + VI) as the between-participants and Animal (positive/approach, threat/avoid) and Time (pre-, post-manipulation) as the within-participant variables. For brevity, only the most pertinent effects are reported. The Animal × Time interaction attained significance, *F*(1,156) = 618.93, *p* < .001, partial *η*^*2*^ = 0.80. Fear beliefs decreased significantly for the positive/approach animal, *t*(159) = 14.24, *p* < .001, *d* = 1.12, and increased significantly for the threat/avoid animal, *t*(159) = −13.59, *p* < .001, *d* = 1.07. These effects were further subsumed within the Animal × Time × Group interaction, *F*(3,156) = 56.19, *p* < .001, partial *η*^*2*^ = 0.52.

To tease apart this interaction we performed a repeated measures ANOVA with Animal (positive/approach, threat/avoid) and Time (pre-, post-manipulation) as the within-participant variables for each group separately. For the AATT group, the Animal × Time interaction revealed a non-significant statistical trend (see Table 2). Fear beliefs decreased significantly for both the approach and avoid training animal (although the effect for the avoid training animal did not survive multiple testing correction where Bonferroni corrected α = .0025).^2^ For the remaining three groups, the Animal × Time interaction attained significance. Fear beliefs decreased significantly for the positive information/approach training animal and increased significantly for the threat information/avoidance training animal.

A change in fear beliefs score computed separately for the positive/approach animal and threat/avoid animal (post score − pre score) was compared across groups. For the positive/approach animal, the magnitude of change in fear beliefs differed significantly between groups (see Table 3). The reduction in fear beliefs was significantly larger for the VI compared to AATT group (although this effect does not survive stringent multiple testing correction where corrected α = .0025) and for the VI + AATT and AATT + VI groups compared to AATT alone. All other comparisons were non-significant. The same pattern of findings, but comparing the magnitude of the increase in fear beliefs was observed for the threat/avoid animal with all comparisons surviving multiple testing corrections (see Table 3).

### 3.3 Effects on implicit attitudes

Trials in which children incorrectly identified the target and trials in which reaction times were greater than 2.5 standard deviations above the individual mean for each condition were excluded (*N* = 340, 4.29%). A mixed ANOVA was performed on reaction times to categorise the word targets with Group (VI, AATT, VI + AATT, AATT + VI) as the between participants factor and Animal (positive/approach, threat/avoid) and Target Valence (positive word, negative word) as the within-participant variables.^3^ The critical Animal × Target Valence (*p* = .888) and Animal × Target Valence × Group (*p* = .553) interactions were non-significant.

### 3.4 Effects on indirect avoidance behaviour

A mixed ANOVA was performed on distance ratings with Group (VI, AATT, VI + AATT, AATT + VI) as the between participants and Animal (positive/approach, threat/avoid) and Time (pre-, post-manipulation) as the within-participant variables.^4^ The Animal × Time interaction attained significance, *F*(1,155) = 177.82, *p* < .001, partial *η*^*2*^ = 0.53. Participants placed themselves significantly nearer to the positive/approach animal (i.e. greater approach tendency) at the post relative to pre-time point, *t*(158) = 7.68, *p* < .001, *d* = 0.61, 197.70 vs. 101.60 and farther away from the threat/avoid animal, *t*(158) = −8.64, *p* < .001, *d* = 0.69, 204.38 vs. 351.53. These effects were further subsumed within the 3-way Animal × Time × Group interaction, *F*(3,155) = 20.01, *p* < .001, partial *η*^*2*^ = 0.28.

To tease apart this interaction, we performed a repeated measures ANOVA with Animal (positive/approach, threat/avoid) and Time (pre-, post-manipulation) as the within-participant variables for each group separately (see Table 2). For the AATT group, the Animal × Time interaction was non-significant. For the remaining three groups the Animal × Time interaction was significant. Distances decreased significantly for the positive/approach animal (however, the effect for the VI only group marginally failed to survive stringent multiple testing correction where corrected α = .0025^5^) and increased significantly for the threat/avoidance animal.

A change score computed separately for the positive/approach animal and threat/avoid animal (post − pre distance) was compared across groups. The magnitude of change in avoidance differed significantly between groups (see Table 3). For the positive/approach animal the decrease in avoidance ratings did not differ significantly for the VI compared to AATT group. The reduction in avoidance was significantly larger for the VI + AATT and AATT + VI groups compared to the AATT group (although the comparison between AATT vs. VI + AATT does not survive multiple testing correction where corrected α = .0025). All other comparisons were non-significant. For the threat/avoid animal, the increase in avoidance ratings was significantly larger for the VI compared to AATT group and for the VI + AATT and AATT + VI groups compared to AATT alone. All other comparisons were non-significant.

### 3.5 Effects on direct avoidance behaviour

**Fig. 3:**
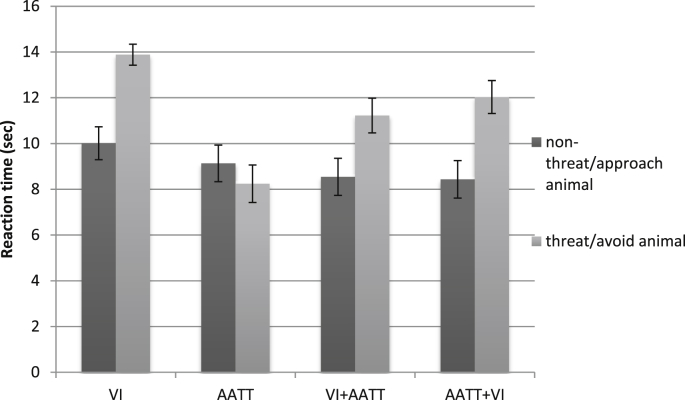
Mean reaction time (sec) to approach the pet carrier (errors bars ± 1 SE).

A 2-way mixed ANOVA was performed on reaction times to perform the behavioural avoidance test with Group (VI, AATT^6^ VI + AATT, AATT + VI) as the between participants and Animal (positive/approach, threat/avoid) as the within-participant variables. The Animal × Group interaction attained significance, *F*(3,155) = 9.52, *p* < .001, partial *η*^*2*^ = 0.16 (see Fig. 3). There was no significant difference in reaction times to approach the approach animal and avoid animal in the AATT group, *t*(38) = 1.26, *p* = .216, *d* = 0.20, 9.13 vs. 8.24. For the remaining groups participants took significantly longer to place their hand in the box containing the threat information/avoid animal than the positive information/approach animal (VI: *t*(39) = −5.21, *p* < .001, *d* = 0.83; VI + AATT: *t*(39) = −4.71, *p* < .001, *d* = 0.74; AATT + VI: *t*(39) = −4.54, *p* < .001, *d* = 0.72).

A reaction time difference score (Avoid/Threat animal − Approach/Positive animal) was compared across groups with a larger value indicative of relatively greater avoidance for the threat/avoid compared to positive/approach animal. The magnitude (and direction) of the difference score differed significantly between groups (see Table 3). This difference score was significantly larger for the VI compared to AATT group and for the VI + AATT and AATT + VI groups compared to the AATT group. These effects survived stringent multiple testing correction where corrected α = .005.^7^ All other comparisons were non-significant.

### 3.6 Supplementary analyses

All analyses were repeated with the between participant factors of which animal (e.g. quoll or quokka) was assigned to be the positive/approach animal or threat/avoid animal and gender. No significant main effects or interactions with these factors were observed and the overall profile of results was unchanged.

## 4 Discussion

Approach-avoidance training was successful in training the desired behavioural responses. Approach movements significantly increased for the animal that was consistently pulled closer and significantly decreased for the animal that was pushed away. The impact of approach-avoidance training on fear-related responses was limited: there were significant reductions in fear beliefs for *both* the approach and avoid animal, but no significant effect on implicit attitudes or avoidance behaviour. Providing children with verbal information had pervasive effects on fear-related responses. Threat information led to a significant increase in fear beliefs and relatively greater avoidance behaviour. Positive information led to a significant decrease in fear beliefs and relatively less avoidance behaviour. No significant effects were observed for implicit attitudes. The pattern of findings for the combined conditions was similar to that observed for verbal information only. We found no convincing evidence for an additive effect of verbal information and approach-avoidance training, irrespective of the order in which they were combined.

Consistent with past research, exposure to verbal threat information appears to be an important pathway for the aetiology of childhood fears (see Muris & Field, 2010 for a review). Effect sizes were consistently larger for threat information than positive information. This could reflect a methodological detail, although the information was matched for content and salience. It could also suggest that verbal information is a more powerful route to inducing fear than reducing it, at least for the present stimuli, which were novel and not of obvious fear relevance. Notwithstanding this, the possibility remains that the provision of positive verbal information may be an effective corrective tool (Kelly et al., 2010). However, the present findings and those of Kelly et al., (2010) demonstrate the effect of only a short burst of non-threat/positive information about stimuli about which children have no or limited prior experience. It is as yet untested how effective positive verbal information might be in modifying long-established fears in clinically anxious children. However, these preliminary results are encouraging as positive information yielded medium to large effects for change in fear beliefs, *d* = 0.92, and indirect avoidance behaviour, *d* = 0.48 (although it is important to note that the effect of positive verbal information on indirect avoidance behaviour marginally failed to survive multiple testing corrections. However, effect sizes are in our opinion of greater value in interpreting the magnitude of effects than merely relying on statistical significance).

We did not fully replicate previous findings showing that approach-avoidance training had differential effects on self-reported fear beliefs. In an initial study (Huijding et al., 2009) no clear effects were seen – when gender was considered, girls but not boys showed greater fear beliefs about the avoided than approached animal. In a second study, a measure of fear beliefs given at post-training showed fear beliefs were higher for the avoided than approached animal (Huijding et al., 2011). In this study, fear beliefs were assessed at pre- and post-training permitting stronger conclusions regarding the effect of approach-avoidance training on *change* in fear beliefs. Fear beliefs decreased significantly for *both* the approach and avoid animal, although the effect size was larger (and in the hypothesised direction) for the approach animal.

We found no convincing evidence that training avoidance tendencies *increased* self-report fear beliefs, which argues against a causal relationship between behavioural response tendencies and fear responses in childhood. However, this conclusion is premature given the infancy of research using the approach-avoidance task and the nature of the stimuli used in the present experiment. Stronger evidence for causality may emerge with more extensive approach-avoidance training. It is perhaps also noteworthy that the AATT lacks a motivational component typically inherent in operant conditioning whereby the behaviour reinforced or minimised leads to a biologically significant event (e.g. repeated training to pull a lever (reinforced behaviour) leads to delivery of food (reward)). Learning the behaviour to approach one animal and avoid the other is not reinforced with a tangible and significant outcome. One possibility is that stronger effects on fear-related responses would be observed if the approach behaviour was rewarded (e.g. pulling the joystick leads to the award of points, or hearing a pleasant sound) and the avoidant behaviour was reinforced by the absence of an aversive experience (e.g. pushing the joystick is reinforced by not losing points, or not hearing an unpleasant sound).

It remains unclear why avoidance training led to *reductions* in fear beliefs (and indirect avoidance behaviour), although these effects were small and didn't survive multiple testing corrections. Notwithstanding this, one possible explanation is that previous studies did not assess pre-post training change in fear beliefs. When examining only post-training fear beliefs data, a similar pattern to that reported by Huijding et al. is observed with fear beliefs (and indirect avoidance ratings) higher for the avoided than approach animal (albeit not significantly so). One possibility is that repeated administration of the avoid animal picture over successive training trials in the absence of overt negative consequences may have attenuated any negative effects of avoidance training on fear beliefs and avoidance behaviour.

Neither verbal information nor approach-avoidance training altered children's implicit attitudes. The affective priming task may not be sensitive enough to detect the effect of the fear manipulations on implicit evaluations. The empirical evidence from adult studies investigating whether training of behavioural responses affects implicit evaluations is mixed (Vandenbosch & De Houwer, 2011; Woud et al., 2011) and no significant effects were reported in past child studies (Huijding et al., 2009, 2011). More extensive approach-avoidance training or the provision of more or repeated verbal information may produce results on an implicit measure. Studies that have demonstrated effects on implicit attitudes have employed considerably more training trials (Kawakami, Phills, Steele, & Dovidio, 2007). The word stimuli used may also have been too distal from the nature of the fear manipulations. Future research should consider using approach (e.g. touch, hold) and avoidance (e.g. hide, run) oriented words or positive (gentle, soft) and negative (angry, dirty) words that are more semantically consistent with the verbal information presented.

This was the first study to compare the effects of verbal information and approach avoidance training and to investigate the combined effects of these two pathways. The effects of approach-avoidance training were consistently smaller compared to verbal information alone or the combined conditions. The only exception being the comparison between VI and AATT conditions for the change in fear beliefs for the positive/approach animal where the effect was no longer significant once stringent multiple testing corrections were applied, but still represented a medium effect size (*d* = 0.48). On balance, our results provide relatively robust evidence that a short burst of verbal information represents a stronger route to fear learning (and fear reduction) about a novel animal in children than does the pairing of a picture of a novel animal with an approach or avoid behavioural response. Unlike previous findings that reported an interaction between verbal information and a negative conditioning experience (Field & Storksen-Coulson, 2007) and a vicarious learning episode (Askew et al., 2008), we did not find an additive effect of combining VI and AATT compared to VI alone. This indicates that expectancies created via verbal information did not act to strengthen the CS-UR association proposed to underpin approach-avoidance training nor strengthen the CR potentially via US revaluation. However, the weak effects of approach-avoidance training may have limited the potential for verbal information to influence the strength of association/response. As previously outlined, it remains unclear the extent to which approach-avoidance training mimics operant conditioning due to the lack of a motivational component in the UR or CR. Given that associative learning may not have occurred in the AATT condition, it is perhaps premature to fully dismiss an associative account in favour of a propositional explanation. Nonetheless, the present findings are more consistent with the idea that conditioned responding is driven by proposition formation rather than association formation (De Houwer, 2009; Mitchell et al., 2009). Verbal information alone is sufficient to create a proposition (e.g. quolls are dangerous) that drives fear-related responses at a cognitive and behavioural level. However, we would still advocate further research into the interaction between different pathways to fear acquisition, as it is highly likely that complex fears originate through multifaceted interactions between different possible pathways to fear learning.

This study has several limitations. First, order of administration of the cognitive and behavioural outcome measures was not counterbalanced, which may have introduced order effects into the data. Second, we observed an imbalance in age between the VI and AATT + VI conditions despite randomisation, which may have biased our statistical tests. It remains possible that the observed differences between these groups on outcome measures may be explained by age and not the effect of the experimental manipulations and that age may interact with the experimental manipulations to influence outcome. However, we consider this unlikely given that all outcome variables were uncorrelated with age and the difference in mean age between the groups was less than 1 year. Third, it is unclear the extent to which children believed that a real animal was present in the BAT. To reduce the possible impact of placing ones hand into the first box and realising that a real animal was not inside, the order of presentation was kept constant to ensure that the first animal (the quoll) that the child was asked to respond to was across participants equally often the animal associated with positive information/approach training and threat information/avoidance training. Fourth, the present study focused on novel, benign looking animals. Larger effects may be observed with fear-relevant stimuli or for familiar animals about which children already possess fear (e.g. spiders, snakes). Finally, the present experiment contrasted positive information/approach and threat information/avoid training limiting specific conclusions regarding independent effects of these conditions. While partly mitigated by the use of pre-post measures, the inclusion of a suitable no-training condition using neutral information or no behavioural response contingencies should be investigated.

### 4.1 Conclusions

Verbal information is a plausible pathway involved in the aetiology of childhood fear learning. This is an important finding because most children are frequently exposed to potentially threatening information, whether that be via peers, parents or through mass media (Muris & Field, 2010). We found only very limited evidence that training approach-avoidance tendencies influenced fear-related responses and no evidence for an additive effect of verbal information and approach-avoidance training. However, from a clinical perspective, exposure therapy for childhood phobias is often highly efficacious and thus the potential role of approach/avoidance in the onset and reduction of childhood fears is worthy of further investigation. Our findings suggest that actively focussing on children's semantic evaluative beliefs regarding feared animals may be a powerful route to fear reduction. In fact, giving positively tinted, realistic information as a way of modifying threat-based interpretations and beliefs is already common during cognitive-behavioural interventions for childhood fears (Ollendick & King, 1998). The extent to which established fears are amenable to change via positive information alone is worthy of further investigation.

## Figures and Tables

**Table 1:** Sample characteristics (standard deviation in parentheses) by condition.

	Entire sample	Condition				Test statistic (df)	*p*
		VI	AATT	VI + AATT	AATT + VI		
Gender (% M)	49	50	48	50	48	χ^2^(3) = 0.10	.99
Age	8.80 (1.13)	8.57 (1.17)	9.02 (1.16)	8.82 (1.04)	9.34 (1.03)	*F*(3,156) = 3.46	.02
Trait anxiety	35.75 (6.87)	37.05 (7.11)	36.13 (6.31)	34.08 (7.01)	36.33 (6.67)	*F*(3,156) = 1.42	.14

**Table 2:** FBQ and NRT mean scores at pre and post time points by Condition (standard deviation in parentheses).

	Condition															
	VI				AATT				VI + AATT				AATT + VI			
	Pre	Post	*F/t* (df)	*d/η*^*2*^	Pre	Post	*F/t* (df)	*d/η*^*2*^	Pre	Post	*F/t* (df)	*d/η*^*2*^	Pre	Post	*F/t* (df)	*d/η*^*2*^
**FBQ**																
Animal × Time			*F*(1,39) = 108.94***	0.74^a^			*F*(1,39) = 3.53^†^	0.08^a^			*F*(1,39) = 371.22***	0.91^a^			*F*(1,39) = 372.37***	0.91^a^
Positive/Approach animal	1.56 (0.64)	0.69 (0.87)	*t*(39) = 5.75***	0.91^b^	1.50 (0.69)	1.03 (0.70)	*t*(39) = 4.40***	0.69^b^	1.48 (0.60)	0.42 (0.44)	*t*(39) = 10.72***	1.70^b^	1.60 (0.81)	0.37 (0.45)	*t*(39) = 10.35***	1.64^b^
Threat/Avoid animal	1.67 (0.77)	3.44 (0.70)	*t*(39) = −11.03***	1.75^b^	1.43 (0.72)	1.20 (0.80)	*t*(39) = 2.35*	0.37^b^	1.51 (0.60)	3.40 (0.60)	*t*(39) = −17.72***	0.37^b^	1.62 (0.78)	3.54 (0.64)	*t*(39) = −11.75***	1.86^b^
**NRT**																
Animal × Time			*F*(1,39) = 64.58***	0.62^a^			*F*(1,39) = 0.01	0.00^a^			*F*(1,38) = 66.69***	0.64^a^			*F*(1,39) = 71.93***	0.65^a^
Positive/Approach animal	201.80 (183.86)	108.05 (170.01)	*t*(39) = 3.04**	0.48^b^	188.53 (141.87)	153.13 (153.88)	*t*(39) = 1.61	0.25^b^	168.69 (115.60)	66.84 (88.47)	*t*(38) = 5.68***	0.91^b^	231.05 (163.78)	77.53 (108.97)	*t*(39) = −6.27***	0.99^b^
Threat/Avoid animal	180.90 (145.02)	443.08 (218.71)	*t*(39) = −7.35***	1.16^b^	207.95 (168.09)	174.93 (184.73)	*t*(39) = 1.55	0.24^b^	184.15 (134.87)	373.56 (185.71)	*t*(38) = −6.57***	1.06^b^	244.00 (194.64)	415.10 (218.02)	*t*(39) = −5.64***	0.89^b^

^†^*p* < .10; *p < .05; **p < .01; ***p < .001. *F* statistics are reported for a repeated measures ANOVA with Animal (positive/approach, threat/avoid) and Time (pre-, post-manipulation) as the within-participant variables for each condition separately. *t* statistics are reported for the comparison between pre and post scores.

a Partial eta squared.

b Cohen's *d.*

**Table 3:** FBQ, NRT and BAT difference scores by condition (standard deviation in parentheses).

	Test statistic (df)	Condition			
		VI	AATT	VI + AATT	AATT + VI
FBQ positive/approach change score	*F*(3,156) = 7.36***	−0.87^A^ (0.95)	−0.47^B^ (0.68)	−1.07^A^ (0.63)	−1.23^A^ (0.75)
FBQ threat/avoid change score	*F*(3,131.90)^a^ = 59.74***	1.78^A^ (1.02)	−0.23^B^ (0.62)	1.90^A^ (0.68)	1.92^A^ (1.03)
NRT positive/approach change score	*F*(3,155) = 3.98**	−93.75^AB^ (194.86)	−35.40^A^ (139.17)	−101.85^B^ (112.00)	−153.53^B^ (154.86)
NRT threat/avoid change score	*F*(3,139.33)^a^ = 18.52***	262.18^A^ (225.76)	−33.03^B^ (134.79)	189.41^A^ (180.03)	171.10^A^ (191.74)
BAT RT difference score	*F*(3,147.70)^a^ = 9.52***	3.87^A^ (4.69)	−0.88^B^ (4.39)	2.68^A^ (3.60)	3.60^A^ (5.00)

*p < .05 **p < .01^;^ ***p < .001. *F* statistics are reported for a univariate one way ANOVA with Condition as a between participant variable. Means with different letters are significantly different (*p* < .05).

a Brown Forsythe corrected df.

## Footnotes

1 In line with past research (Field & Lawson, 2003), the order of presentation was kept constant for both the NRT and BAT to ensure that the first animal (the Quoll) the child was asked to respond to/approach was across participants equally often the animal associated with positive information/approach training and threat information/avoidance training. There were no significant differences in RTs or distance away from the animal for participants who approached the positive information/approach trained animal first relative to those who approached the positive information/approach animal second (*p*'s > .05).

2 For the FBQ, multiple testing corrections were applied using a stringent Bonferroni-corrected alpha level of .0025. This corrects for the analyses performed to compare fear belief scores for the positive/approach and threat/avoid animal within each condition (see Table 2) and for the contrasts performed comparing difference scores across the four conditions for the positive/approach and threat/avoid animal (see Table 3).

3 Data were unavailable for twelve participants due to time constraints while testing or difficulties in the child being able to read the words.

4 Data were unavailable for one participant on this task within the VI + AATT condition.

5 For the NRT, multiple testing corrections were applied using a stringent Bonferroni-corrected alpha level of .0025. This corrects for the analyses performed to compare avoidance measurements for the positive/approach and threat/avoid animal within each condition (see Table 2) and for the contrasts performed comparing difference scores across the four conditions for the positive/approach and threat/avoid animal (see Table 3).

6 Data were unavailable for one participant in AATT condition due to time constraints.

7 For the BAT, multiple testing corrections were applied using a stringent Bonferroni-corrected alpha level of .005. This corrects for the analyses performed to compare reaction times for the positive/approach compared to threat/avoid animal within each condition and for the contrasts performed comparing difference scores across the four conditions (see Table 3).
